# Core Elements, Development, and Implementation Strategies of the Nursing Minimum Data Set: Scoping Review

**DOI:** 10.2196/84281

**Published:** 2026-02-27

**Authors:** Pascal Müller, Annabell Gangnus, Katrin Gayen, Uta Kirchner-Heklau, Patrick Jahn, Sebastian Hofstetter

**Affiliations:** 1 Health Service Research Working Group, Acute Care, Department of Internal Medicine, Faculty of Medicine, University Medicine Halle (Saale), Martin-Luther-University Halle-Wittenberg, Magdeburger Straße 12, Halle (Saale), 06112, Germany, 49 3455574001; 2 Dorothea-Erxleben-Learning-Centre, Faculty of Medicine, University Medicine Halle (Saale), Martin-Luther-University Halle-Wittenberg, Halle (Saale), Germany

**Keywords:** Nursing Minimum Data Set, standardized nursing data, nursing informatics, nursing documentation, core nursing data elements, health information systems, digital health

## Abstract

**Background:**

Nursing care systems face significant challenges due to demographic changes, a workforce shortage, and rising demand for care services. Digital assistive technologies offer potential to address these challenges, but systematic and standardized nursing data are essential to evaluate both innovations and broader care processes. The Nursing Minimum Data Set (NMDS) provides a foundational framework for capturing structured information on nursing care, yet there is no international consensus on its core content, development, and practical use.

**Objective:**

This scoping review aims to map current international literature regarding (1) the core content elements of NMDS, (2) methodological approaches used in NMDS development, and (3) implementation and use of NMDS in different nursing settings.

**Methods:**

Following the JBI (Joanna Briggs Institute) methodology and Arksey and O’Malley framework, a systematic search was conducted on July 2, 2025, in the MEDLINE (via PubMed) and CINAHL (via EBSCO) databases using the term “nursing minimum data set.” Inclusion was restricted to studies in English or German focusing on the content, development, or implementation of the NMDS. The research team reviewed studies in an independent and double-blinded fashion for eligibility based on predefined criteria, with discrepancies resolved by consensus. Eligible studies were narratively summarized, with extraction structured into categories based on the review’s research questions.

**Results:**

From 1908 initially identified articles, 26 (1.4%) studies met the inclusion criteria. Considerable heterogeneity was found in the structure and scope of the NMDS, with datasets comprising 16 to 145 items. Despite variation, 4 central domains consistently emerged: patient demographics, medical care information, nursing care elements, and institutional or organizational data. NMDS development typically followed a participatory, multistage approach involving literature analysis, stakeholder consensus building, and validation through pretesting and real-world application. Implementation and use of the NMDS serve multiple functions, including documenting nursing care processes, supporting workload measurement and resource planning, quality assurance, benchmarking, and demonstrating nursing’s contribution to patient outcomes. However, successful implementation depends on technical, legal, organizational, and educational strategies. Core challenges include a lack of standardized terminology, inconsistent legal frameworks, and varying levels of staff training and acceptance.

**Conclusions:**

The NMDS provides a robust basis for standardized nursing documentation, quality assurance, and health system planning, but international variability and ongoing challenges in harmonization, integration, and acceptability persist. Advancing the NMDS requires collaborative efforts for interoperability, investment in digital infrastructure, and targeted education. Further research should focus on comparative effectiveness, cross-context validation, and strategies to reduce documentation burden while maximizing data utility.

## Introduction

The nursing care system in Germany is confronted with significant challenges, including demographic shifts, a shortage of nursing personnel, and increasing demand for care services [1]. From 2014 to 2021, expenditures on long-term care insurance increased more than 2-fold, rising from €25.5 billion (US $30 billion) to €53.9 billion (US $63.4 billion) [2]. To ensure the sustainability of care provision, there is a need for innovative digital solutions. Digital assistive technologies have been shown to have considerable potential in promoting participation and autonomy, as well as reducing care dependency [3]. However, research focusing on the etiology and progression of care needs, as well as the ramifications of digital innovations, has been disproportionately underrepresented to date [4].

Robust and standardized nursing data are essential to evaluate these innovations and their impact on care. The Nursing Minimum Data Set (NMDS) is defined as “a minimum set of items of information with uniform definitions and categories concerning the specific dimension of nursing which meets the information needs of multiple data users in the health care system” [5]. The NMDS provides a structured basis for capturing nursing care across populations, settings, and regions. It facilitates the monitoring of trends in care delivery and resource use, serves as a foundational element for research by establishing connections with broader health information systems, and contributes to the formulation of health policy, thereby ensuring the visibility of nursing contributions in health care planning and decision-making [6]. With a focus on influencing factors and opportunities to prevent or reduce care dependency, the NMDS can also serve as a framework for assessing the impact of digital innovations.

Despite its potential, there is a lack of clarity regarding which elements should be included in a core nursing dataset, how such elements are developed, and what barriers or success factors influence its implementation in practice. The objective of this scoping review is to map the current state of the literature by addressing the following research questions:

1. Which content elements comprise a nursing minimum dataset?
2. Which methodological approaches have been used in its development?
3. What is the implementation and use of such a dataset in different nursing settings?

## Methods

This scoping review was conducted in accordance with the JBI (Joanna Briggs Institute) methodology [7] and guided by the framework of Arksey and O’Malley [8], which structures the process into the following phases: identifying relevant studies, study selection, data presentation, and synthesis of results. To enhance rigor and transparency, the reporting process adhered to the PRISMA-ScR checklist (Preferred Reporting Items for Systematic Reviews and Meta-Analyses extension for Scoping Reviews; Checklist 1) [9].

### Search Strategy

A systematic search was conducted on July 2, 2025, in the MEDLINE (via PubMed) and CINAHL (via EBSCO) databases using the search term “nursing minimum data set,” applied identically in both databases. “Nursing minimum data set” is an internationally established and unambiguous term for the concept of interest. Preliminary searches using broader terms such as “nursing data” or “nursing data set” yielded numerous irrelevant results, mostly referring to general medical datasets including nursing-related variables but not specifically designed for nursing care. Searches in other databases (eg, Google Scholar and Europe PMC) produced even more irrelevant hits.

Using the precise term “nursing minimum data set” maximized specificity and relevance. PubMed’s Automatic Term Mapping further captured synonyms and MeSH (Medical Subject Headings) terms. This focused approach aligns with Freguia et al [10] but extends their work by also analyzing the development and implementation of the NMDS. The MEDLINE and CINAHL databases were selected due to their comprehensive coverage of peer-reviewed nursing and health services research. No date restrictions were applied, and reference lists of the included articles were manually screened to minimize the risk of missing eligible studies.

The detailed inclusion and exclusion criteria are enumerated in Textbox 1. Exclusion criteria were based on our aim to map the conceptual, methodological, and implementation aspects of the NMDS rather than secondary applications or specialized derivatives. Studies focusing solely on specific clinical conditions, measurement tools, isolated settings (eg, palliative care), or context (eg, quality management) were excluded. Publications on other datasets (eg, the Minimum Data Set Resident Assessment Instrument [11]) or nursing classifications were also excluded. Language was restricted to English and German to ensure reliable screening and data extraction. Although this may introduce language bias, the majority of conceptual NMDS literature is published in these languages.

Textbox 1. **Inclusion and exclusion criteria.**

**Inclusion criteria**

- Primary and secondary studies with abstracts available
- Language: German or English
- Description of content, development, implementation, and use in practical applications of the Nursing Minimum Data Set

**Exclusion criteria**

- Secondary analyses of the Nursing Minimum Data Set
- Reference to interventions, specific clinical pictures, or medications
- Reference to quality management, personnel indicators, or specific settings
- Comparison of measurement instruments
- In cases of duplicate publication, only the most comprehensive or earliest version was included to avoid double-counting

### Study Selection and Data Extraction

The study selection followed the JBI methodology for transparency and reproducibility. Duplicate removal and screening were conducted using Rayyan (Rayyan Systems, Inc) [12], a web-based collaborative platform enabling independent and blinded review. Duplicates were first removed automatically using the software’s matching algorithms based on title, author, DOI, journal, and year and then manually checked to resolve partial matches or inconsistent metadata.

Titles and abstracts of remaining records were independently screened by 2 reviewers (PM and AG) using Rayyan’s blinded mode. Records identified as potentially eligible by at least 1 reviewer proceeded to full-text review. Discrepancies were resolved through discussion, with a third reviewer (KG) consulted if consensus could not be reached. The process was independent of study design and focused on studies addressing the conceptualization, development, or implementation of the NMDS. The number of records at each stage is presented in the PRISMA (Preferred Reporting Items for Systematic Reviews and Meta-Analyses) flow diagram. Reference lists of the included studies were manually screened to identify further eligible studies.

To systematically extract and present relevant information, included studies were narratively summarized in a data-charting form. The studies were categorized and analyzed according to the following classifications: “overview” (eg, definition and elements); “development” (eg, methods and processes); and “implementation” (eg, evaluation, availability, reliability, and validity). The primary categories were derived deductively from the review questions. Subcategories were subject to refinement and expansion in an iterative manner as novel themes and patterns emerged from the included studies. This framework enabled the synthesis of structured and flexible approaches, capturing the breadth of the research field.

## Results

### Study Selection

**Figure 1.**
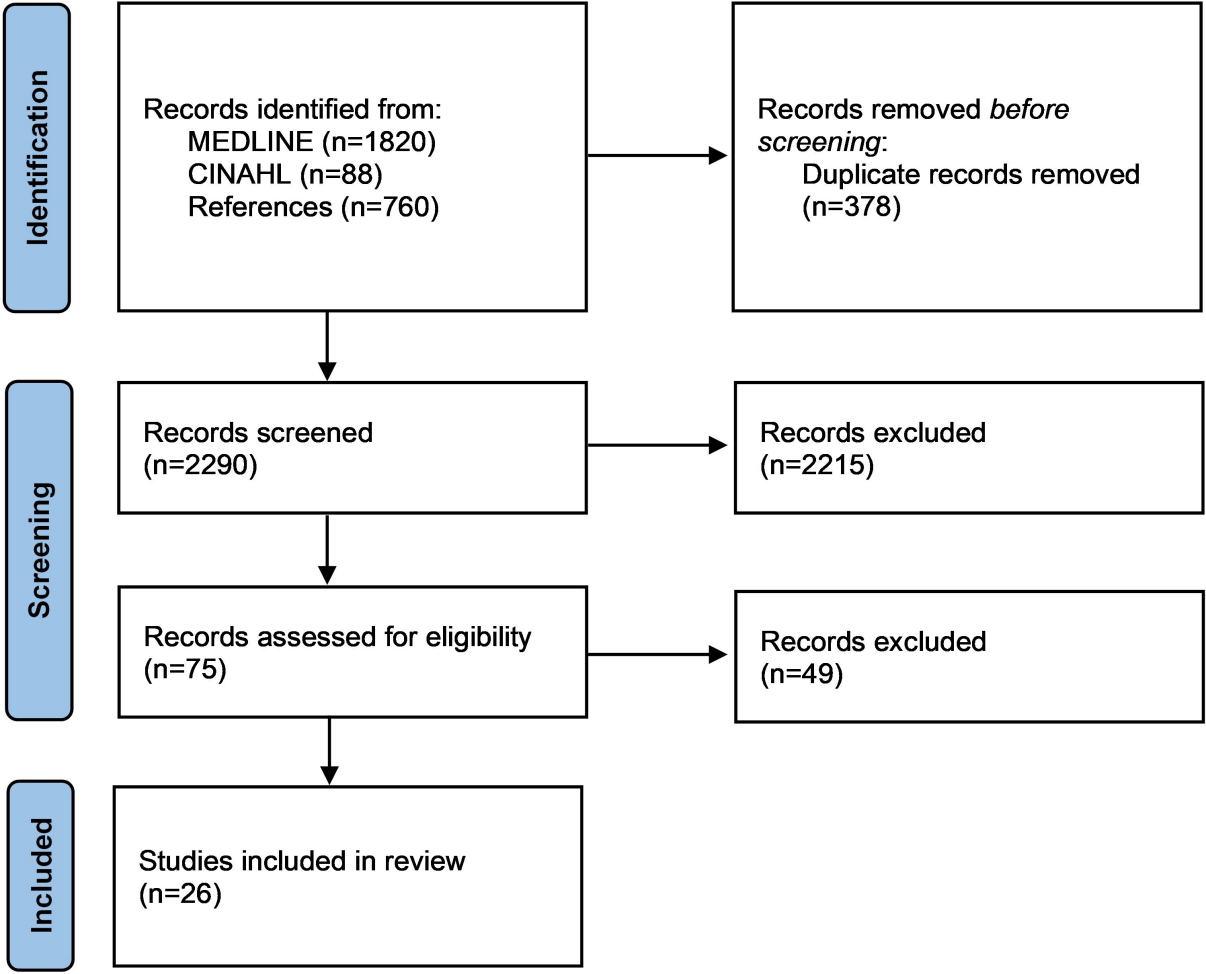
PRISMA (Preferred Reporting Items for Systematic Reviews and Meta-Analyses) flowchart illustrating the study selection process.

The database search conducted in July 2025 yielded 1908 results (PubMed: n=1820, 95.4%; CINAHL: n=88, 4.6%). After removing duplicates, 1869 (N=1908, 98%) records remained for title and abstract screening. Of these, 1823 (N=1869, 98%) were excluded due to unavailability of full texts or because they focused on data infrastructure, storage, or evaluation frameworks unrelated to NMDS content, development, or implementation. A total of 36 (N=1869, 2%) articles underwent double-blind full-text review. After applying all eligibility criteria, 23 (N=1869, 1.2%) articles were included. Screening reference lists added 3 more studies, resulting in a total of 26 included studies. No conflicts occurred among independent reviewers. The selection process is presented in the PRISMA flowchart [13] (Figure 1). A comprehensive list of the included studies and the data extraction table are provided in Multimedia Appendix 1 [6,10,14-37].

### Main Characteristics of the Included Studies

The 26 studies originated from 9 countries. Most were from the Netherlands (n=6, 23.1%), the United States (n=4, 15.4%), Austria (n=4, 15.4%), Belgium (n=4, 15.4%), and Ireland (n=3, 11.5%). Additional studies were conducted in Finland (n=2, 7.7%), and 1 (3.8%) each from Italy, Germany, and Thailand. The majority of reported NMDS initiatives have their origins in the United States [10,16,19,25,29], Belgium [10,19,25,29,32-34,36], Canada [10,19,25,29], Thailand [10,25,27,29,37], Switzerland [25,29], and the Netherlands [18,20-23,25,29]. Additional origins including Ireland [14,26,29], Germany [17,29], Finland [24,25,29,35], Australia [19,25,29], Sweden [25], Brazil [25], Japan [25], Austria [28,30,31], and Scotland [19] were mentioned in several studies, which also referenced a European [10,19,25] and an international NMDS [19,25]. Most of the studies focused on universal nursing settings. However, there have also been investigations of specific settings such as hospitals, long-term care facilities, nursing homes, home health care agencies, and public health contexts.

The articles encompassed a period from 1988 to 2022. The predominant language of publication was English (n=24, 92.3%), with 2 (7.7%) articles published in German. The study designs exhibited heterogeneity, encompassing theoretical papers (n=9, 34.6%), mixed methods studies (n=7, 26.9%), quantitative studies (n=9, 34.6%), and a single qualitative study. The predominant focus was the development of the NMDS [14,17,21,27,28,30,33-36], followed by evaluation [16,20,22,31] and implementation [24,26,32]. A subset of these studies was also partially cited in the included systematic review [29] and umbrella review [10].

### Data Elements

The datasets have been designed to capture comprehensive and comparable information on nursing practice, patient status, and care context. This facilitates the monitoring, evaluation, and enhancement of nursing care delivery at both the individual and system levels. A review of the existing literature revealed a considerable variation in the number of elements comprising an NMDS, ranging from 16 to 145 items per dataset. Despite this variation, 4 key categories consistently emerged: patient demographics, medical care information, core nursing care elements, and institutional or organizational data (Textbox 2).

The patient’s demographic information is typically documented on an initial admission to the health care facility and subsequently serves as a foundational reference point for the patient’s characteristics. The medical care items in question prioritize crucial medical information. While certain elements are documented on a single occasion during the patient’s hospital stay, details concerning medical procedures and medications are frequently recorded throughout the patient’s stay. Core nursing care elements are typically collected continuously throughout the duration of hospitalization. The exception to this is nursing outcomes, which are generally assessed at discharge. Risk assessments and nursing diagnoses, on the other hand, are updated as the patient’s status changes. Institutional or organizational data link patient- and care-level information with structural and organizational attributes, thereby supporting contextual analysis of care processes.

Textbox 2. **The datasets are designed to capture information on nursing practice, patient status, and care context.**

**Patient demographics (3-8 items)**

- Personal identification (pseudonymized)
- Date of birth and age
- Gender
- Race and ethnicity
- Place of residence
- Address and phone number

**Medical care information (4-7 items)**

- *International Classification of Diseases* codes
- Medical history (patient and family)
- Drug allergies or other allergies
- Procedures
- Laboratory tests
- Medication
- Dead or alive codes and resuscitation status
- Admission or encounter date
- Discharge information (date and summary)
- Length and progression of stay
- Health status (stability, severity, complications, and critical events)

**Nursing care elements**

- Nursing diagnosis (17-48 items)

- Nursing interventions (8-170 items)
- Nursing outcome (4-40 items)
- Intensity of nursing care (3-4 items)
- Nursing process (10 items)
- Client status (activities of daily living)
- Discharge plan
- Care planning (goals of the shift and goals of nursing care)
- Medical instruments’ use before discharge

**Institutional or organizational data**

- Health care setting (4-5 items): type of institution, main point of service, type of nursing delivery system, and other disposition point

- Resource allocation (4 items): number and qualification of available nurses, number of nursing hours available, number of beds, and nurse-to-patient ratios
- Institutional or provider identifiers: service or agency number, health record number, nurse identifier, and physician identifier
- Expected payer of the bills or health insurance

### NMDS Development

The development of the NMDS generally follows a three-stage process: (1) selection and definition of relevant data elements based on literature analysis, document analysis, and interdisciplinary consensus; (2) validation and consensus building through expert rounds, pretesting, and reliability assessments; and (3) application and testing the feasibility in real-world settings.

### Selection and Definition of Data Elements

Potential items were chosen for clinical relevance and feasibility through literature reviews, analysis of clinical records (n=15-45 per study), and collaborative expert panels or Delphi processes. Participants included field nurses, nurse managers, nursing scientists, informatics specialists, and policy representatives (n=22-287 per study). Semistructured interviews and questionnaires were central, supported by focus groups and multiple rounds of expert panels (up to 11 with n=5-88 participants). The goal was to ensure that the items reflected actual practice, captured the diversity of nursing activities, and could be coded in a feasible manner. Critical decisions included choosing terminology and coding schemes, unit of data collection (eg, patient day or episode of care), and timing of data entry.

### Refinement and Validation

The refinement of items and structures was achieved through consensus-building methods, such as repeated Delphi rounds and focus groups, along with pretests and reliability assessments, including Cohen κ and intraclass correlation coefficient. These methods often incorporated interdisciplinary and practice-based feedback, facilitating the continuous improvement and refinement of the research instruments. In the preliminary phase, the expert participants engaged in deliberations concerning the relevance, frequency, and practical feasibility of each item. In addition, they addressed the conceptualization, frequency, and relevance of the items, along with the data that were routinely collected. Lists of nursing diagnoses, interventions, and outcomes were developed and discussed for clarity and practicality. Subsequent rounds entailed the refinement and evaluation of the wording and definitions for their usefulness and feasibility, as well as the systematic testing of the definitions and coding rules. The resulting data collection instruments provided multiple response options for each item, thereby generating a broad range of analyzable variables. Each data element was carefully defined and accompanied by specific operational instructions, including scoring schemes that frequently used 5-point Likert scales to evaluate interventions and diagnoses.

The documentation of nursing care is typically implemented through the use of standardized classification systems. However, a universal system has yet to be established across all NMDS implementations. Instead, a variety of established frameworks are used, including the International Classification of Nursing Practice [38], the North American Nursing Diagnosis Association-I classification [39], the Omaha System [40], and the Home Health Care Classification [41], among others. The NMDS has been designed to either specify a preferred system or permit cross-mapping between local and international standards. The ability to exchange data and information seamlessly across different systems and between different countries is paramount for the comparability of data at both the national and international levels.

Reliability and validity testing constituted a fundamental component of the second stage. Pretests were conducted using samples ranging from 4 to 214 participants, often comparing the extracted information from patient records (n=20 to nearly n=300,000) by pairs of raters (research staff and clinical nurses). The coding agreement was subsequently quantified. The validity of the content was determined through its alignment with existing literature and user evaluations.

### Feasibility Testing and Application

The final development stage focused on evaluating the usability and practical implementation of the NMDS. This included structured pilot applications in real nursing settings, such as hospitals, nursing homes, ambulatory care, and home health agencies. Typically, a longitudinal design with repeated measurements over a defined observation period (eg, 7 d or 1 admission episode) was selected. Consequently, the NMDS demonstrated its efficacy in the domains of care process description, workload measurement, resource planning support, benchmarking, and quality improvement across diverse nursing settings.

One such function is to systematically describe the diversity of nursing care and patient populations through frequency analyses of nursing diagnoses, interventions, and outcomes. The application of advanced analytical approaches, such as relative to an identified distribution analysis, has been demonstrated to facilitate the illustration of variations in nursing practice and complexity across a variety of settings, age groups, and medical diagnoses. In addition, the NMDS is instrumental in evaluating the intensity of nursing care. By establishing a correlation between the implementation of each nursing intervention and the temporal and experiential parameters of the nursing personnel involved, health care institutions can facilitate a quantitative assessment of the workload associated with each patient or unit of care. Classification systems such as the nursing diagnosis index or patient dependency models (eg, the San Joaquin classification) are used to establish a correlation between patient needs and staffing requirements, thereby facilitating evidence-based staffing decisions and resource allocation.

A further central benefit of the NMDS is its ability to demonstrate nursing’s contribution to patient outcomes. For instance, path analysis can be used to examine the relationship between nursing interventions and patient progress, as illustrated by improvements in functional indices (eg, the Barthel index) during hospitalization. In the context of quality management and patient safety, outcome indicators facilitate the identification of care problems and the evaluation of improvement strategies through the integration of validated assessment instruments. The longitudinal use of these tools adds further value by enabling the tracking of care trends and patient outcomes over extended periods. These analyses are valuable for clinical managers and policymakers in the context of epidemiological analysis, benchmarking institutions, highlighting areas of best practice, and supporting the dissemination of evidence-based interventions. This facilitates the formulation of strategic decisions regarding care processes and resource distribution.

A comprehensive implementation strategy must encompass not only technical solutions, such as increasing automation and electronic data collection, but also legal, organizational, and educational strategies. For instance, the implementation of the NMDS in specific contexts was terminated due to the absence of a legal foundation or inadequate institutional endorsement. The quality and utility of collected data are contingent on nurses’ familiarity with the NMDS content and vocabulary. Educational initiatives are required to enhance nurses’ comprehension of the significance of structured data collection and its pertinence for clinical decision-making, quality assurance, and the development of interoperable hospital information systems. To reduce the workload associated with NMDS documentation, it may be necessary to minimize the scope of collected variables, with a push toward electronic and automated data collection.

## Discussion

### Principal Findings

This scoping review offers a comprehensive mapping of the extant literature on the NMDS with regard to content elements, methodological development processes, and implementation and use across nursing settings. Synthesizing evidence from diverse international contexts and research designs, this review addresses the critical knowledge gaps identified in the introduction and provides an informed basis for further research and policy development in the field of standardized nursing data.

A salient finding of this review is the substantial heterogeneity observed in the structure and length of reported NMDSs, which vary from 16 to 145 items. Nevertheless, 4 core domains consistently emerged: patient demographics, medical care information, nursing care elements, and institutional or organizational data. This convergence on key domains, despite variation in the level of detail and specific items, underscores both the complexity of capturing nursing care and the international consensus on the essential pillars of nursing-relevant information. The comprehensive integration of nursing diagnoses, interventions, and outcomes signifies a worldwide transition toward outcome-oriented, evidence-based nursing practice. However, the absence of a universally adopted classification system for these categories—variously using systems such as the North American Nursing Diagnosis Association-I classification, the International Classification of Nursing Practice, or local taxonomies—continues to challenge the comparability of nursing data both within and across countries. This diversity underscores the necessity for enhanced harmonization to facilitate broader benchmarking, interoperability, and secondary data use.

The development of the NMDS is characterized by rigorous, iterative, and participatory processes. Most of the studies used a 3-stage approach: first, systematic identification and selection of content elements (frequently drawing from the literature, expert consultations, and clinical record analyses); second, consensus building and validation (frequently relying on Delphi processes, focus groups, and pretesting); and third, pilot implementation in real-world settings. The consistent engagement of field nurses, managers, scientific experts, and policy stakeholders supports both the clinical relevance and the practical feasibility of the resulting datasets. However, methodological diversity—particularly in the degree of stakeholder inclusion, the criteria for item selection, and the approach to validation—limits the extent to which the results can be compared and generalized. A fundamental finding is that consensus and interdisciplinary involvement significantly enhance acceptance and operationalization. However, disparities in contextual adaptation and infrastructure availability necessitate context-sensitive approaches, superseding the notion of a universally optimal solution.

The review demonstrates a wide range of applications of the NMDS, encompassing care process documentation, workload measurement, resource planning, quality assurance, benchmarking, and outcome evaluation. Analytical techniques such as frequency analysis, relative to an identified distribution analysis, and path analysis facilitate the sophisticated use of NMDS data. This includes the identification of care trends and variations, the mapping of nursing workload, and the demonstration of the impact of nursing care on patient outcomes. NMDS data have demonstrated their relevance for supporting clinical management, health policy, and epidemiological surveillance in multiple health care systems.

However, the implementation of NMDS is not without challenges. Achieving success in this endeavor necessitates the implementation of a multifaceted approach, encompassing not only technical solutions such as electronic and automated data collection but also legal, organizational, and particularly educational strategies. In certain settings, the discontinuation of NMDS initiatives was attributed to a lack of legal foundation, inadequate institutional support, or suboptimal integration into health information systems. The collection of consistent and high-quality data is contingent upon nurses’ familiarity with the terminology and documentation standards. Consequently, training and change management are imperative. Furthermore, the balancing of data comprehensiveness with feasibility, as well as the minimization of documentation burden, emerges as a recurring theme in the pursuit of long-term sustainability.

### Strengths and Limitations

A central strength of this review is its comprehensive and systematic approach, which adheres to established methodological frameworks (JBI and PRISMA-ScR) and uses a transparent, replicable search and data extraction process. The program’s emphasis on international literature and a diverse array of study types contributes to the generalizability and relevance of the findings. The use of independent, blinded screening and iterative data charting enhances the rigor and credibility of the synthesis.

Several limitations of this review should be acknowledged. First, only 2 databases and a single search term were used. Preliminary scoping checks indicated that broader terms or additional databases yielded mostly irrelevant results, suggesting a low likelihood of missing studies specifically addressing the NMDS. As NMDS initiatives are sometimes embedded in national health IT programs, relevant reports may appear in gray literature or regional journals not indexed in the MEDLINE or CINAHL databases. Nevertheless, the potential remains that studies using alternative terminology or published outside the MEDLINE and CINAHL databases were not captured. This structural publication bias should be considered when interpreting the findings. Second, the exclusion criteria may limit the mapping breadth by underrepresenting NMDS adaptations in non–English-speaking and non–German-speaking countries or highly specialized settings. This potential exclusion could introduce publication bias and limit the capture of ongoing or context-specific innovations. Third, as is typical for scoping reviews, no formal quality appraisal of included studies was conducted, which may have affected the weight assigned to individual findings. Fourth, the heterogeneity of study designs, settings, and health care systems complicates a direct comparison and synthesis of findings. To conclude, while this review identifies fundamental content and process elements, as well as prevalent barriers and success factors, it does not provide a meta-analytic estimate of the impact of the NMDS on patient or system outcomes.

### Conclusions

This scoping review highlights the heterogeneity and evolving consensus on the structure, development, and implementation of the NMDS. Despite international variations, 4 core domains consistently emerged: patient demographics, medical care information, nursing care elements, and institutional or organizational data. The NMDS can enhance the visibility, quality, and effectiveness of nursing care across health care systems, provided that challenges related to standardization, interoperability, and acceptance are addressed.

To translate these findings into practice, policymakers and health care managers should prioritize the standardization of nursing documentation through appropriate legal frameworks, investment in digital infrastructure, and targeted educational initiatives for nursing staff. Practical steps include establishing interoperable digital nursing records; providing structured training on NMDS use; and implementing pilot programs to assess the feasibility, usability, and data utility. Researchers are encouraged to evaluate the NMDS across diverse settings, conduct comparative effectiveness studies, and explore strategies to reduce documentation burden while maximizing actionable insights for care management, resource planning, and policy decisions. Overall, the review underscores that advancing the NMDS requires interdisciplinary collaboration, context-sensitive implementation, and ongoing evaluation to ensure that standardized nursing data meaningfully inform clinical practice and health system planning.

## Supplementary material

10.2196/84281 Multimedia Appendix 1 Data extraction sheet.

10.2196/84281 Checklist 1 PRISMA-ScR checklist.

## Abbreviations

JBI: Joanna Briggs Institute
MeSH: Medical Subject Headings
NMDS: Nursing Minimum Data Set
PRISMA: Preferred Reporting Items for Systematic Reviews and Meta-Analyses
PRISMA-ScR: Preferred Reporting Items for Systematic Reviews and Meta-Analyses extension for Scoping Reviews

## References

[1] Behrendt S, Tsiasioti C, Schwinger A, Schwinger A, Kuhlmey A, Greß S, Klauber J, Jacobs K, Behrendt S (2024). Pflege-Report 2024.

[2] Rothgang H, Müller R (2021). Wirkungen der Pflegereformen und Zukunftstrends.

[3] (2024). Assistive technology. World Health Organization.

[4] Blüher S, Stein T, Schilling R, Grittner U, Kuhlmey A, Jacobs K, Kuhlmey A, Greß S, Klauber J, Schwinger A (2021). Pflege-Report 2021.

[5] Werley HH, Lang NM (1988). Identification of the Nursing Minimum Data Set.

[6] Werley HH, Devine EC, Zorn CR, Ryan P, Westra BL (1991). The Nursing Minimum Data Set: abstraction tool for standardized, comparable, essential data. Am J Public Health.

[7] von Elm E, Schreiber G, Haupt CC (2019). Methodische anleitung für scoping reviews (JBI-methodologie). Z Evid Fortbild Qual Gesundhwes.

[8] Arksey H, O’Malley L (2005). Scoping studies: towards a methodological framework. Int J Soc Res Methodol.

[9] Tricco AC, Lillie E, Zarin W (2018). PRISMA extension for scoping reviews (PRISMA-ScR): checklist and explanation. Ann Intern Med.

[10] Freguia F, Danielis M, Moreale R, Palese A (2022). Nursing minimum data sets: findings from an umbrella review. Health Informatics J.

[11] (2025). Long-term care facility resident assessment instrument 3.0 user’s manual. Centers for Medicare & Medicaid Services.

[12] Hirt J, Nordhausen T (2019). Digitale anwendungen für die studienauswahl im rahmen von systematischen evidenzsynthesen. Pflege.

[13] Moher D, Liberati A, Tetzlaff J, Altman DG, PRISMA Group (2009). Preferred reporting items for systematic reviews and meta-analyses: the PRISMA statement. PLoS Med.

[14] Butler M, Treacy M, Scott A (2006). Towards a nursing minimum data set for Ireland: making Irish nursing visible. J Adv Nurs.

[15] Coenen A, Schoneman D (1995). The nursing minimum data set: use in the quality process. J Nurs Care Qual.

[16] Devine EC, Werley HH (1988). Test of the nursing minimum data set: availability of data and reliability. Res Nurs Health.

[17] Eberl I, Bartholomeyczik S (2010). The Belgian Nursing Minimum Data Set II (B-NMDS II) and its transfer to German hospitals: results of the first investigation phase, the translation and adaption process of the instrument. Pflege.

[18] Goossen WT (2001). Exploiting the Nursing Minimum Data Set for the Netherlands. Stud Health Technol Inform.

[19] Goossen WT, Epping PJ, Feuth T, Dassen TW, Hasman A, van den Heuvel WJ (1998). A comparison of nursing minimal data sets. J Am Med Inform Assoc.

[20] Goossen WT, Epping PJ, Feuth T, van den Heuvel WJ, Hasman A, Dassen TW (2001). Using the Nursing Minimum Data Set for the Netherlands (NMDSN) to illustrate differences in patient populations and variations in nursing activities. Int J Nurs Stud.

[21] Goossen WT, Epping PJ, Van den Heuvel WJ, Feuth T, Frederiks CM, Hasman A (2000). Development of the Nursing Minimum Data Set for the Netherlands (NMDSN): identification of categories and items. J Adv Nurs.

[22] Goossen W, Dassen T, Dijkstra A, Hasman A, Tiesinga L, van den Heuvel W (2003). Validity and reliability of the Nursing Minimum Data Set for the Netherlands (NMDSN). Scand J Caring Sci.

[23] Goossen WTF (2002). Statistical analysis of the Nursing Minimum Data Set for The Netherlands. Int J Med Inform.

[24] Kaakinen P, Torppa K (2009). Implementation of a structured nursing documentation in a special care unit. Stud Health Technol Inform.

[25] Mac Neela P, Scott PA, Treacy MP, Hyde A (2006). Nursing minimum data sets: a conceptual analysis and review. Nurs Inq.

[26] Morris R, Matthews A, Scott AP (2014). Validity, reliability and utility of the Irish Nursing Minimum Data Set for General Nursing in investigating the effectiveness of nursing interventions in a general nursing setting: a repeated measures design. Int J Nurs Stud.

[27] Phuphaibul R (2006). Nursing data set development in Thailand. Stud Health Technol Inform.

[28] Ranegger R, Hackl WO, Ammenwerth E (2014). A proposal for an Austrian Nursing Minimum Data Set (NMDS): a Delphi study. Appl Clin Inform.

[29] Ranegger R, Ammenwerth E (2014). Nursing Minimum Data Sets (NMDS) - a literature review relating to objective and data elements. Pflege.

[30] Ranegger R, Hackl WO, Ammenwerth E (2015). Development of the Austrian Nursing Minimum Data Set (NMDS-AT): the third Delphi round, a quantitative online survey. Stud Health Technol Inform.

[31] Ranegger R, Hackl WO, Ammenwerth E (2015). Implementation of the Austrian Nursing Minimum Data Set (NMDS-AT): a feasibility study. BMC Med Inform Decis Mak.

[32] Sermeus W, Delesie L, Van den Heede K, Diya L, Lesaffre E (2008). Measuring the intensity of nursing care: making use of the Belgian Nursing Minimum Data Set. Int J Nurs Stud.

[33] Sermeus W, Van den Heede K, Michiels D (2004). A nation-wide project for the revision of the Belgian Nursing Minimum Dataset: from concept to implementation. Stud Health Technol Inform.

[34] Sermeus W, van den Heede K, Michiels D (2005). Revising the Belgian Nursing Minimum Dataset: from concept to implementation. Int J Med Inform.

[35] Turtiainen AM, Kinnunen J, Sermeus W, Nyberg T (2000). The cross-cultural adaptation of the Belgium Nursing Minimum Data Set to Finnish nursing. J Nurs Manag.

[36] Van den Heede K, Michiels D, Thonon O, Sermeus W (2009). Using nursing interventions classification as a framework to revise the Belgian nursing minimum data set. Int J Nurs Terminol Classif.

[37] Volrathongchai K, Delaney CW, Phuphaibul R (2003). Nursing Minimum Data Set development and implementation in Thailand. J Adv Nurs.

[38] Hinz M, Dörre F, König P, Tackenberg P (2003). ICNP: Internationale Klassifikation für die Pflegepraxis.

[39] Herdman TH, Kamitsuru S, Lopes C (2025). NANDA-I-Pflegediagnosen: Definitionen und Klassifikation 2024-2026.

[40] Martin KS, Norris J (1996). The Omaha System: a model for describing practice. Holist Nurs Pract.

[41] Saba VK (2002). Nursing classifications: Home Health Care Classification System (HHCC): an overview. Online J Issues Nurs.

